# Time to death among HIV-infected under-five children after initiation of anti-retroviral therapy and its predictors in Oromiya liyu zone, Amhara region, Ethiopia: a retrospective cohort study

**DOI:** 10.1186/s12887-021-03072-6

**Published:** 2022-01-03

**Authors:** Bereka Tefera Marie, Weldemariam Sintayehu Argaw, Bitewa Yibelu Bazezew

**Affiliations:** 1 Lecturer, Midwifery Department, Kotebe Metropolitan University, Addis Ababa, Ethiopia; 2 Public Health Emergency Management Officer, Bati District Health Office, Bati, Ethiopia; 3 Lecturer, Midwifery Department, Debremarkos University, Debre Markos, Ethiopia

**Keywords:** Antiretroviral therapy,under Five Children, Death, Northeast, Ethiopia

## Abstract

**Background:**

Human Immunodeficiency Virus (HIV) is infection which mainly attacks immune system of an individual. Its disease progress is rapid in children and if treatment is not initiated nearly half of infected children will die by the second year of infection. In Ethiopia, nearly twenty four percent of HIV related death is happen to under-five children; however studies done in this specific age group are limited are with poor evidence of predictors.

**Objectives:**

To determine time to death and identify predictors of death in HIV infected under- five children on antiretroviral therapy in Amhara regional state, Oromia ‘liyu’ zone, Northeast Ethiopia, from 2014 to 2019.

**Methods:**

Institution based retrospective follow up study was conducted in 376 under-five HIV- infected children on antiretroviral therapy from January 2014 to December 2019 in health institutions in Oromia Liyu Zone, Amhara region, Ethiopia. Multivariable Cox-proportional hazard regression model was used to identify independent predictors of mortality in HIV- infected under-five children on antiretroviral therapy.

**Result:**

At the end of follow up, 304 (80.85%) of HIV-infected children were alive, 39 (10.95%) were lost to follow up, 12 (3.19%) were transferred out and 21 (5.59%) were reported dead due to HIV/AIDS. The cumulative survival probabilities of children after 3, 6, 12, 24 and 36 months were 0.99, 0.98, 0.97, 0.89 and 0.87 respectively. The overall mean time to death was 19.7 months (95%CI = 18.74–20.67) with incidence of 5.9 deaths per 100 child-months (95%CI: 3.89–9.09). Children with severe malnutrition at baseline (AHR = 4.9; 95 CI: 1.04, 23.50), advanced WHO clinical stage at enrolment (AHR = 3.9; CI: 1.37, 10.88), poor adherence to ART (AHR = 6.56; CI: 3.33, 10.14) and with no history of Isoniazide prophylaxis were significantly associated to higher mortality events (AHR = 3.6; CI: 1.24, 10.18).

**Conclusion:**

Death of HIV-infected under-five children on ART is high within the first one year after enrolment. The risk of death increased if the child was malnourished at beginning of treatment, had poor ART adherence, with advanced WHO clinical stages and lack of Isoniazide prophylaxis during their age of infancy.

## Background

Human Immunodeficiency Virus (HIV) is a viral infection which mainly attacks immune system. The disease progress is rapid in under-five children, and if left without treatment nearly half of they would be dead by the second year of infection. Strides made in HIV treatment have impacted disease progression and immune capacity, which continue to improve survival rates and quality of life which is also evidenced in Ethiopia [1–3]. The natural history and clinical manifestations of HIV in children differs from adults. Rapid disease progress seen in infants and the opportunistic infections that result from immune deficiency in under-five children with HIV are often primary, not reactivation disease, thereby resulting in greater morbidity. Worldwide HIV/AIDS accounted for 1.4% under-five children deaths in 2015 and over 120,000 children died due to AIDS-related illnesses in 2016, this equates to 328 deaths every day [4]. Even though 62% reduction in AIDS-related deaths and 70% decline in new HIV since 2000, still the burden is visibly high. For instance it seems great achievement to decline new infection from 490,000 in 2000 to 150,000 in 2015, but the magnitude unacceptably high [5–8]. In Ethiopia estimated 722,248 people are living with HIV with prevalence of 1.16% in a general population and children accounts for around ten percent of overall infection. According to 2017 report of CDC, HIV/AIDS is the 7^th^ cause of death in Ethiopia affecting children at high rate [4]. Moreover, twenty four percent of all AIDS-related deaths are among under-five children, more than thirty percent of people with new infections are children, and ART coverage for children is around 34%, below the national target of having 85 percent of children living with HIV on ART by 2020. In 2018 PMTCT coverage, which is assumed to be major preventive factor for under-five child deaths, was still in between 63 to 93 percent, which needs high effort. Coverage of early infant diagnosis of HIV is estimated at around seventy percent and in 2019 from all causes of under-five mortality HIV/AIDS accounts for 2% [1, 3, 9]. Although visible efforts aimed at improving HIV infected child survival have driven large reductions in mortality levels among under- five children, presence of persistent and intolerably high numbers of child and young adolescent deaths mean more work remains to be done to address the specific survival needs of children and young adolescents in Ethiopia [10, 11]. This study is aimed at addressing survival status and magnitude of deaths in HIV infected under-five children receiving ART by identifying significant predictors that have role in mortalities of these age groups by including more proxy variable and segmented to specific age group of under-five than under 15 children unlike some studies.

## Methods and materials

Study design and setting: Multi facility based institution based retrospective cohort study, was conducted to children who initiated ART on the period from 2014 to 2019 in Amhara regional state, Oromia ‘liyu’ zone, Northeast Ethiopia from March 1, 2020 to April 30 2020. Open cohort was used to a required number of samples in the first four year, include according to zonal administrative report, the current estimated number of population is around 585, 968, majority of the society are farmers, the zone contains seven “weredas” and 127 “kebeles”, which are small administrative units in the region (107 rural and 20 urban). There are two hospitals and twenty-eight health centers, among them more than 90% of them give PMTCT services and the two hospitals and eight of health centers are giving ART services. Currently there around 3, 061 ART taking patients [12].

## Study population

All under-five children with HIV infection and taking ART in government health facilities providing ART service on the zone were used as source population. And under five children who started treatment on the period from 2014 to 2019 were study population. Children with incomplete information on predictors; when one of main independent variables (age of the child, age at the start of ART and regimen) are not present in the document were excluded from the study.

## Sample size determination and sampling technique

Sample size was determined by using statistical STATA version 14, considering proportional allocation of events in log rank test (freed man principles). Significant predictors of mortality among HIV infected under-five children taking ART were used from different literatures and software calculation computed with considering 95% CI, 80% power, 5% level of significance and 10% assumption of withdrawals giving total sample size of 376 for this study. All ten health facilities providing ART service were included and a total under five children on treatment on the period from January 2014 to June 2019 were extracted from the registry. At this stage sampling frame was developed and total sample size was compared with the total under five children on ART and all subsequent patient were included since total number of children on ART in the indicated period were 389.

## Data collection technique and tool

The data collection was open cohort for the first four years to enroll the required sample size in the study. Data was collected from February 1,2020 to March 30,2020 by reviewing the patients’ medical charts (follow up and ART intake forms) using data extraction checklist adapted from ART follow up form and different reviewed published studies. Data on death of HIV positive children while on ART were obtained from providers report on the medical cards. Death at home, after discharge, had been ascertained by the drug adherence counselor (case managers) using contact addresses. The most recent laboratory results before ART initiation were used as baseline values. If there was no pre- treatment laboratory test, results obtained within three month of ART initiation was considered as baseline values.

## Study variables

Time to death due to HIV/AIDS after initiation of ART is the dependent variable. Socio demographic predictors ( as age of child, sex of child, sex of care giver, age of caregiver, marital status of caregiver, Residence of care giver, place of birth, family status of the child) and Clinical and Immunological predictors (Age at start of ART, WHO clinical stage, TB at baseline, Hemoglobin level, CD4 count, Cotrimoxalzole status, INH status, ART regimen, child adherence, nutritional status, co morbid illness, history of recurrent common childhood infections) are independent variable of the study.

## Operational definitions

Advanced WHO clinical stages: are stage III and IV baseline clinical stages of HIV-infected under-five children during enrolment to ART [13].

Anemia: Hemoglobin level is below 10 mg/dl [14].

Censored: When the child is lost-to-follow up, transferred out, withdrawn from follow-up, death by another causes and lives beyond the study time [14].

CD4 count below threshold: CD4 counts less than 350 cells/mm^3^ [15].

Event: death due to HIV infection

Fair adherence to medications: adherence level of under-five children to ART medications within the last three months, between 90-95% was considered as fair adherence [3].

Loss to follow-up: children recorded as died due to non HIV related cause, re located to another district without transferring there treatment and follow-up chart or recorded failed to attend per appointments schedule (who left the follow-up) permanently are lost to follow-up.

Mild WHO clinical stages: are stage I and II baseline clinical stages of HIV-infected under-five children during ART enrolment [13].

Moderate malnutrition: Nutritional anthropometry of weight for height is between 70%-80% or height for age Z-score of less than -3 SD or having weight for age Z-score of < -3 SD [16].

Opportunistic infections: HIV-infected under-five child on ART developed one or more registered history of opportunistic infections during follow-up periods.

Poor adherence to medications: adherence level of under-five children to ART medications within the last three months, less than 90% was considered as poor adherence [3].

Recurrent common child hood infection: Having more than three consecutive episodes of diarrhea, pneumonia, upper respiratory tract infections or skin infections.

Severe malnutrition: when nutritional anthropometry of weight for height is less than 70% or height for age Z-score of less than -3 SD or having weight for age Z-score of < -3 SD [16].

Survival time: the length of time in months a child was followed from the time the child started ART until death, was lost to follow up, or was still on follow up [14].

Data management and analysis: The data was coded and entered into Epi-data version 3.1 and exported to Statistical software package (STATA version 14). Checking for missing values and presence of influential outliers has been done; outliers were managed by taking highest or second smallest values within the data as described by published article on management of missing values and outliers by Kwak SK & Kim JH [17]. Descriptive statistics such as mean (standard deviation) for normally distributed data, median (inter quartile range), frequencies and proportions was used to describe the cohort. Kaplan-Meier survival curve together with log rank test was used to assess survival experience of an individual at specific times and to compare survival between different independent variables. Schoenfeld residuals test was used to check interaction of each covariate with time and graphical methods was used to check the Cox Proportional Hazard (PH) assumption. Uni-variable Cox proportional hazards regression was done for each independent variable and outcome of interest to identify potentially significant variables for consideration in the multivariate Cox proportional hazards regression model. Variables whose significance test below 0.25 in uni-variable analysis had been selected for final model. In addition, context and findings of previous studies were considered in the identification of candidate variables for multivariate analysis. The result of the final model was expressed in terms of adjusted hazard ratio (AHR) with 95% confidence intervals (CI) with significance of P< 0.05. The final model goodness fitness was assessed by using cox-snell residual technique.

## Result

### Baseline socio-demographic characteristics of participants

A total of 376 under-five children were participated in the study. Nearly half of them, 189 (50.3%) were male, 243 (64.63%) of children live in rural areas and the median age of Participants at ART initiation was 32 (IQR= 22-44) months (Table1). Based on the study a huge Segment of under five were delivered at home168 (44.68%).

**Table 1:** Socio-demographic characteristics of HIV infected under-five age children on ART in Oromia ‘liyu’ Zone, Amhara region, Ethiopia 2020

Variables	Category	Frequency (%)
**Age at start of ART** **(in months)**	≤ 24 months	95 (25.27%)
	25–59 months	281 (74.73%)
**Sex**	Male	189 (50.27%)
	Female	187 (49.73%)
**Place of birth of the child**	Health institution	208 (55.32%)
	Home	168 (44.68%)
**Family status**	Both alive	239 (63.56%)
	Father died	61 (16.22%)
	Mother died	30 (7.98%)
	Orphaned	46 (12.23%)
**Sex of care giver**	Male	91 (24.20%)
	Female	285 (75.80%)
**Age of care giver**	< 20 years	6 (1.60%)
	20–35 years	162 (43.09%)
	> 35 years	208 (55.32%)
**Place of residence**	Urban	133 (35.37%)
	Rural	243 (64.63%)
**Marital status of care giver**	Married	256 (68.09%)
	Single	36 (9.57%)
	Divorced	37 (9.84%)
	Widowed	47 (12.58%)

### Baseline clinical characteristics

One hundred fifty (39.89%) of HIV infected under five children born from mothers who do not initiated PMTCT service during their period of pregnancy. Based on WHO clinical staging, 92 (24.47%) of them initiated ART at advanced stage (clinical stage III and stage IV) (Table 2). One hundred ninety nine (52.93%) had normal nutritional status, whereas 138 (36.97%) and 38 (10.11%) of them had either moderate or severe malnutrition respectively. During the study follow up period 280 (74.47%) and 68 (18.09%) of participants have good and fair adherence, respectively; in other side 28 (7.45%) of them were with poor adherence, of them 9 (32%) of them had died at the end of the study. The baseline CD4 count were available for more than three fourth (299 (79.5%)) of under-five children, around 68 (22.74%) of them had CD4 level less than threshold (<350 cells/mm^3^) and remaining 231 (77.26%) of them had CD4 level above threshold (>350 cells/mm^3^) (Table 2).

**Table 2:** Baseline clinical characteristics of under-five children on ART in Oromia ‘liyu’ Zone, Amhara region, Ethiopia 2020

Variable	Category	Frequency (%)
**Maternal history of PMTCT**	Yes	226 (60.11%)
	No	150 (39.89%)
**Baseline Nutritional status**	Severe malnutrition	38 (10.11%)
	Moderate malnutrition	138 (36.97%)
	Normal nutritional status	199 (52.93%)
**History of OI’s during follow up**	Yes	150 (39.89%)
	No	226 (60.11%)
**Adherence status (child)**	Good	280 (74.47%)
	Fair	68 (18.09%)
	Poor	28 (7.45%)
**Baseline WHO staging**	Mild stages	284 (75.53%)
	Advanced stages	92 (24.47%)
**Baseline CD4 count**	Below threshold	68 (22.74%)
	Above threshold	231 (77.26%)
**Baseline hemoglobin**	< 11 mg/dl	109 (28.99%)
	≥ 11 mg/dl	267 (71.01%)
**Cotrimoxalzole prophylaxis**	Yes	258 (68.62%)
	No	118 (31.38%)
**INH prophylaxis**	Yes	226 (60.11%)
	No	150 (39.89%)

### Survival characteristics after initiation of ART

After initiation of ART, HIV-infected children were followed for 3 months to 41 months and median follow up period was 19 months (IQR= 11-26.8). At the end of follow up period, 304 (80.85%) of the children were alive, 39 (10.37%) were lost to follow up, 12 (3.19%) were transferred out to other health facilities, and 21 (5.59%) were reported dead due to HIV/AIDS. Of the 21 deaths, two (9.5%) of them occurred within the first three months after initiation of HAART and 9 (42.85%) of them occurred within the first 12 months of ART initiation. The incidence of death was 5.9 deaths per 100 child-months (95%CI: 3.89-9.09) during the follow-up period. The overall mean time to death was 19.7 months (95%CI= 18.74-20.67). The median estimated survival time of under-five age children after initiation of ART in the study was 19 months (IQR= 11.00-26.80). The cumulative probability of survival of under-five children on ART after last month of follow-up was 87.23% (95%CI; 79.42-92.22). The cumulative survival probabilities of children after 3, 6, 12, 24 and 36 months were 0.99, 0.98, 0.97, 0.89 and 0.87 respectively.

### Comparison of survival curves

**Fig. 1:**
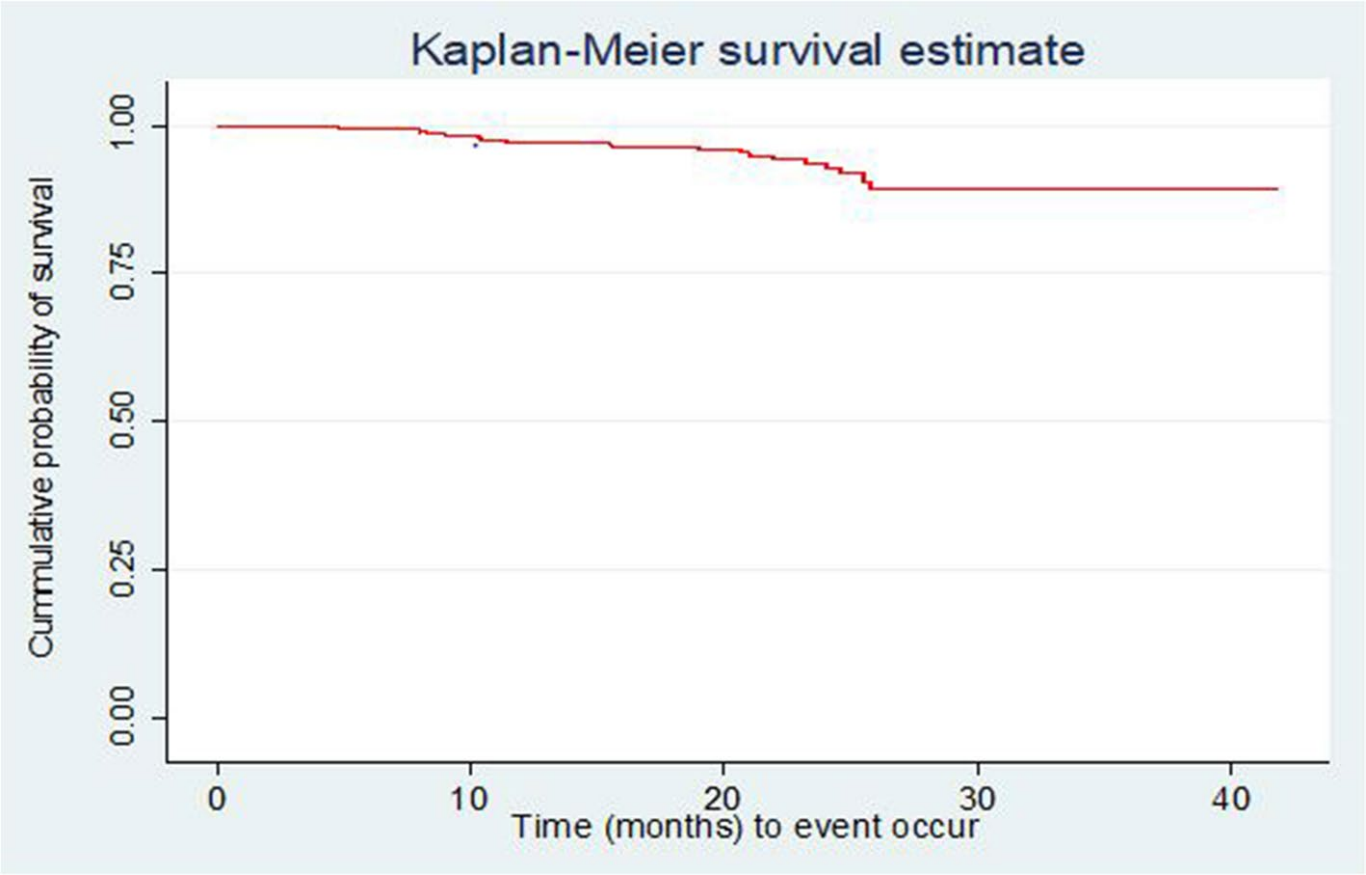
Kaplan-Meier survivor function among under-five children on ART in Oromia 'liyu' Zone, Amhara region, Ethiopia 2020

**Fig. 2:**
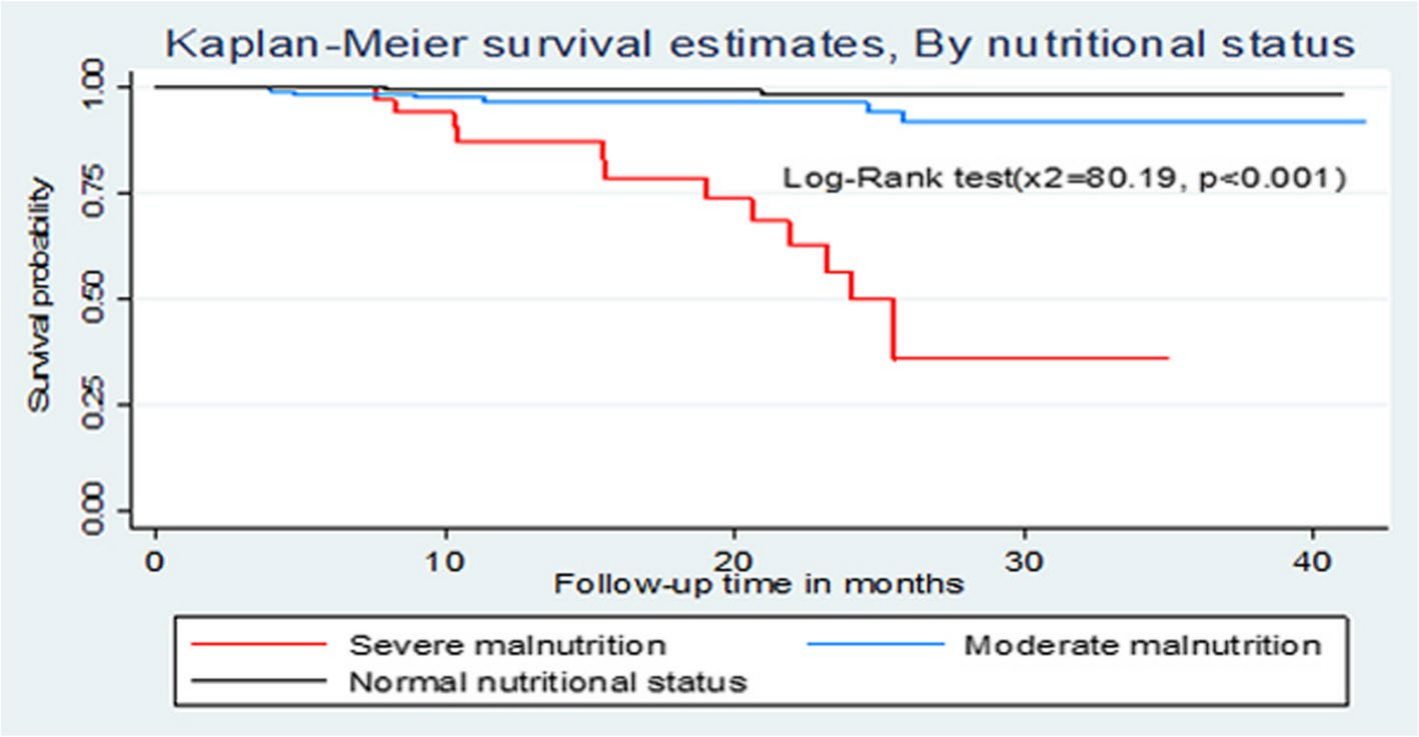
Kaplan-Meier survival estimates of baseline nutritional status among under-five children on ART in Oromia 'liyu' Zone, Amhara region, Ethiopia 2020

**Fig. 3:**
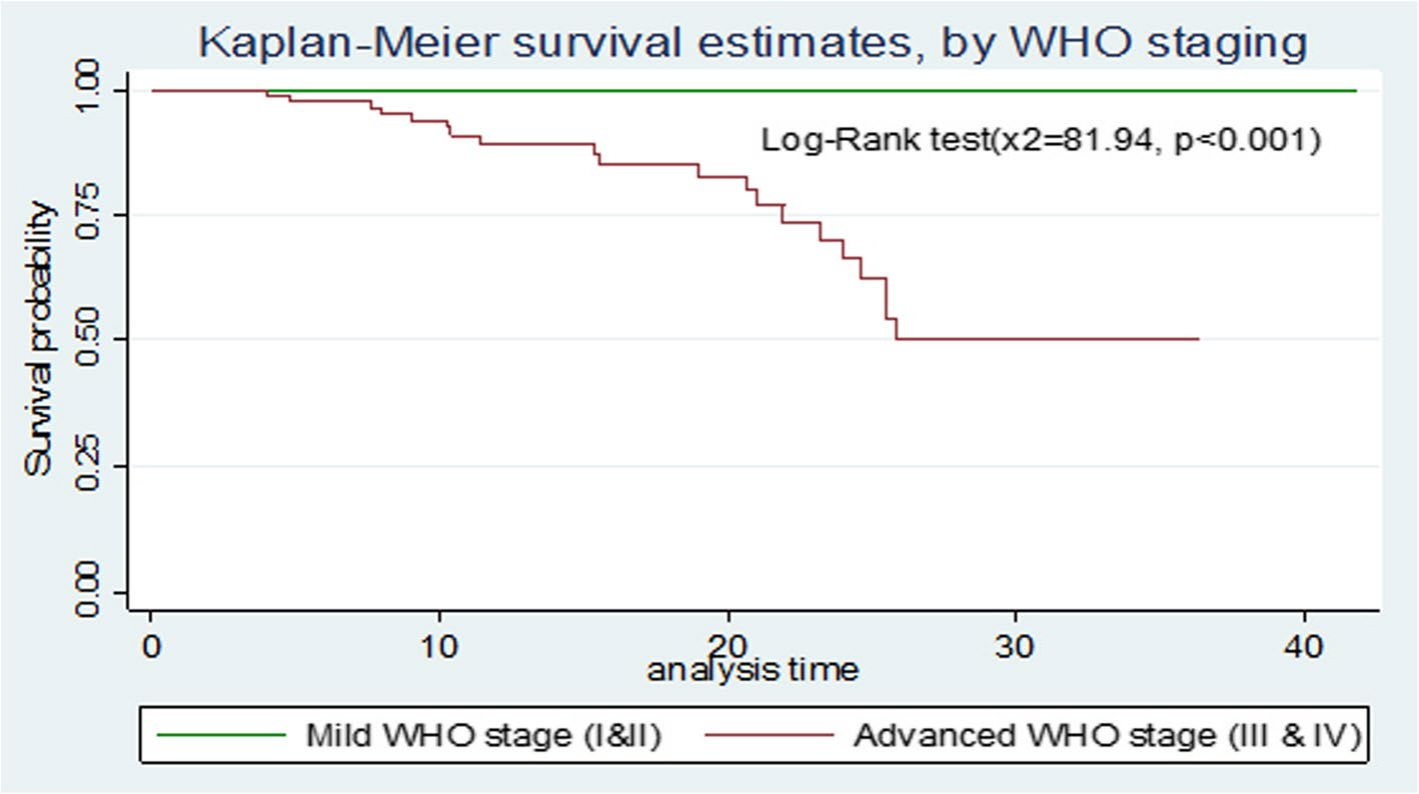
Kaplan-Meier survival estimates of baseline WHO staging among under-five children on ART in Oromia 'liyu' Zone, Amhara region, Ethiopia 2020

**Fig. 4:**
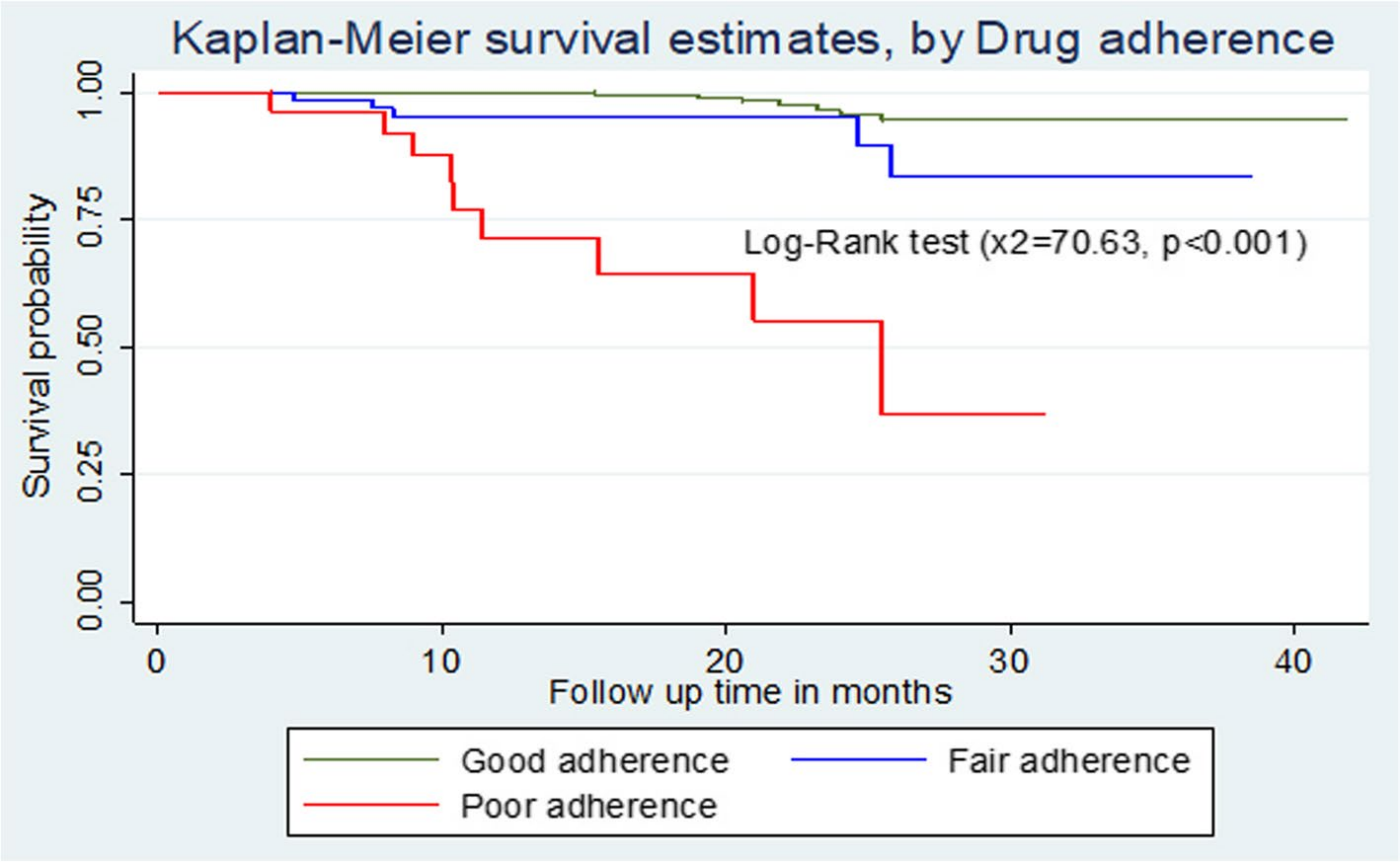
Kaplan-Meier survival estimates based on adherence status among under-five children on ART in Oromia 'liyu' Zone, Amhara region, Ethiopia 2020

**Fig. 5:**
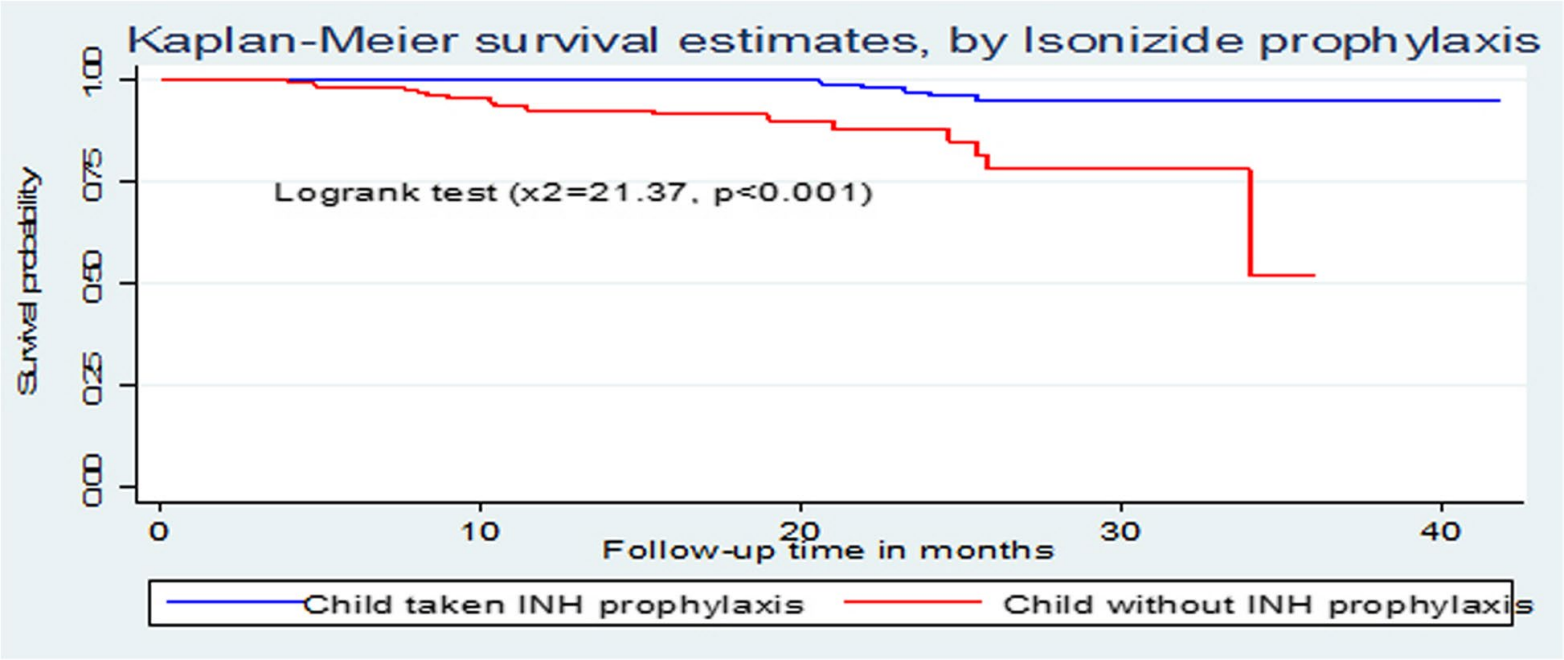
Kaplan-Meier survival estimates based on their Isoniazide prophylaxis among under-five children on ART in Oromia 'liyu' Zone, Amhara region, Ethiopia 2020

The overall Kaplan-Meier survivor function estimate shows that most deaths had been occurred in the earlier months of ART initiation. Among a total of 21 deaths, 2(9.5%) of them occurred in the first six months of follow-up and around 9(42.9%) deaths occurred in the first 12 months of follow-up, which became declining through follow up time and continues steadily at later months of follow up. Some graphs compared to other covariates have shown relatively larger gaps between categories, such as drug adherence, baseline nutritional status and WHO clinical stages of the children (Fig. 1). Based on the results from Kaplan-Meier survival curve, significant covariates (Baseline WHO stage, Drug adherence and nutritional status of the child) have shown certain pattern in outcome event (death) of under-five children throughout the follow up time (Fig. 1). Under-five children with severe malnutrition during initiation of ART have developed death in early months of follow up significantly compared to normal and moderate malnutrition. Children with WHO advanced clinical stage (III and IV) have survived less than children with mild stages (I and II), this observed difference was also statistically significant (*p *< 0.001). Children with good adherence have longer survival experience than those children with fair and poor adherence and those under-five children with poor adherence have lower survival experience compared to fair once, these differences have been statistically significant (*p *< 0.001) (Figs. 2, 3, 4 and 5). The log-rank value of *p *< 0.001 suggests that there was a difference in probability of developing death between groups throughout the follow-up period. The Kaplan-Meier survival curve remarkably decreases for children with severe malnutrition (Figs. 2, 3, 4 and 5).

### Predictors of HIV-infected under-five age child death

Variables whose *p* value less than 0.25 on uni-variable cox-regression analysis were recruited for determination of association in multivariable Cox-regression with level of significance < 0.25. Baseline nutritional status of the child, Child adherence to ART medications during follow up, Baseline clinical stage of the child during ART initiation and status of INH-prophylaxis after birth were identified as independent determinants of death in under-five children. The nutritional status of HIV-infected under-five children was found to be predictors of death, in which the risk of death at any given time during the study period among under-five children with severe malnutrition was almost five (AHR=4.93; 95 CI: 1.04, 10.47) times higher than those children who had normal nutritional status (Table 3). The risk of death among under-five children with poor adherence to ART medications is almost four (AHR=3.9; CI: 1.37, 10.88) times higher than those children with good adherence. In addition, the risk of death among under-five children with advanced WHO clinical stages (III and IV) at enrolment to ART was almost seven times (AHR=6.56; CI: 3.33, 12.14) higher hazard of death than children with mild stages (I and II). In other hand, the risk of death in under-five children without history of INH prophylaxis during their age of infancy was four times (AHR=3.6; CI: 1.24, 10.18) higher than those children with INH prophylaxis.

**Table 3:** Multivariable Cox-regression analysis of predictors of death among under-five children on ART at Amahara regional state, Oromia “liyu” zone, Northeast Ethiopia 2020

Covariates	Categories	Outcome		CHR	AHR	CI (95%)	*P*-values
		**Dead**	**Censored**				
**Baseline nutritional status**	Normal nutritional status	2 (1.11%)	197 (98.99%)	1	1		
	Moderate malnutrition	6 (4.32%)	133 (95.68%)	4.79	2.07	[0.41, 10.39]	0.376
	Severe malnutrition	13 (34.21%)	25 (65.79%)	9.78	4.93	[1.04, 10.47]	0.043
**Adherence status of the child**	Good	7 (2.50%)	273 (97.50%)	1	1		
	Fair	5 (7.35%)	63 (92.65%)	3.56	2.25	[0.70, 7.30]	0.175
	Poor	9 (32.14%)	19 (67.86%)	12.84	3.86	[1.37, 10.88]	0.011
**Baseline WHO Clinical stages**	Mild (stage I & II)	1 (0.35%)	283 (99.65%)	1	1		
	Advanced (stage III&IV)	20 (21.74%)	72 (78.26%)	25	6.37	[3.33, 10.14]	0.002
**INH prophylaxis**	Yes	5 (2.21%)	221 (97.79%)	1	1		
	No	16 (10.67%)	134 (89.33%)	7.99	3.55	[1.24, 10.18]	0.018

## Discussion

In this study, high number of deaths 9(42.9%) occurred within the first one year, after starting ART. This finding is similar to the studies conducted in Ethiopia, Harar [15], Gondar, Addis Ababa [18] Debretabor and Dessie [13], Nigeria [19], Cameroon [20] and study in Sub-Saharan Africa [21], reporting that the death rate was high in early times of ART initiation. Higher death in this period of time could be resulted from almost same health service coverage and late utilization of ART services in the countries. One of significant associated predictors of death among HIV-infected under-five children on ART was baseline nutritional status of the child during enrolment, by which children with severe malnutrition status at beginning ART showed to have shorter duration of survival than the normal once (AHR=4.9; 95 CI: 1.04, 23.52). This finding is consistent with studies done in Ethiopia (Amhara region, Debretabor and Dessie) [13, 16]. This similarity might be due to the fact that children with poor nutritional status may clinically manifest poor outcomes to treatments, since their immunity is compromised by lack sufficient nutrients to their cells; In other hand study in Cameroon explained poor nutritional status is not a cause for increased rate of HIV-infected children [20] this might be because of difference in socio-economic and nutritional culture between the two countries. WHO clinical stage during enrolment to ART was other covariates that had significant effect on mortality of under-five child. The hazard of death on those children with advanced stages (stage III and IV) at ART enrolment was around six times higher than those with mild stage (stage I and II). This finding was consistent with studies done in Ethiopia, Amahra region [16], Gondar [18] and Addis Ababa [22] and the finding is also consistent with studies done in Cameroon [20] and southern Africa [23]. This similarity might be because of children with higher stages have weaker immune status and are highly vulnerable to OIs that may lead to early occurrence of death. In this study from total 92 children with advanced stages at ART enrolment 65 (70.7%) of them had developed one or more OIs during follow up period and from total of 21 deaths during the study period 19 (90.5%) had developed death. In contrast, a study done in Southern regional state (Gamo-Gofa) [14] and Amhara regional state (Desse and Debretabor Hspitaals) [13], on children survival after initiation of ART found that WHO clinical stages as not a predictor of HIV-infected child death. The reason for this difference might be level of community awareness towards early utilization of ART services as effectiveness decreases in late initiation.

Adherence status of the child to medications throughout the follow-up time is important predictor, which is in line with majority of studies. INH prophylaxis status under-five children during their age of infancy were among one of significant determinants of death. Children with no history of INH prophylaxis were associated with reduced survival. This finding was consistent with findings in a study conducted in Gamo-Gofa (Ethiopia) [14]. The cause for this might be due to reduced level of early identification of HIV-infected children and low coverage of PMTCT in between this two places and the whole country (Ethiopia). The possible reason for such higher level of death on these under-five children without INH prophylaxis during ART enrolment could be due occurrence of some OIs like pneumocystis pneumonia, toxoplasmosis, tuberculosis and recurrent diarrheal diseases. In contrast a study done in Addis Ababa on treatment outcomes of HIV-infected children [24] showed that taking INH prophylaxis during has no significant effect on the mortality of ART under-five children, this might be due to difference in accessibility, availability and utilization of health institutions in Addis Ababa and Gamo-Gofa.

## Conclusion

The incidence of death is 5.9 deaths per 100 child-months and the overall mean time to death among under five children on ART in this study is 19.7 months (95%CI= 18.74-20.67). The cumulative probability of survival of under-five children on ART after last month of follow-up was 87.23% i.e. nearly the indicated percentage of children on ART can pass and celebrate the five year of life. The major factors that determine time to death of under-five children with HIV/AIDS on ART were; Baseline nutritional status, WHO clinical stage of the child during ART enrolment, adherence status of the child to medications and INH prophylaxis during infancy period of the child.

### Limitation of the study

As the study relies on retrospective review of data some important variables which can have potential to affect the treatment outcome were not included.

## Abbreviations

3TC: Lamuvidine
AIC: Akaike-Information Criteria
AHR: Adjusted Hazard Ratio
AIDS: Acquired Immune Deficiency Syndrome
ART: Anti Retroviral Therapy
AZT: Zidovudine
CDC: Communicable Diseases Control
CHR: Crude Hazard Ratio
EFV: Efavirenz
GC: Gregorian-Calendar
HAART: Highly Active Anti Retroviral Therapy
HIV: Human Immune Deficiency
INH: Iso Nicotinyl Hydrazide
MTCT: Maternal to Child Transmission
NVP: Neverapine
OI: Opportunistic Infections
PPSS: Pediatric Psycho Social Support
PH: Proportional Hazard
PMTCT: Prevention of Mother To Child Transmission
SDG: Sustainable Development Goal
TB: Tuberculosis
TDF: Tenofovir
UNAIDS: United Nations Program on Acquired Immune Deficiency Syndromes
VIF: Variance Inflation Factor
WHO: World Health Organization
