# Correction to: Time to death among HIV-infected under-five children after initiation of anti-retroviral therapy and its predictors in Oromiya liyu zone, Amhara region, Ethiopia: a retrospective cohort study

**DOI:** 10.1186/s12887-022-03295-1

**Published:** 2022-05-10

**Authors:** Sintayehu Argaw Weldemariam, Zewdu Dagnew, Yilkal Tafere, Tefera Marie Bereka, Yibelu Bazezew Bitewa

**Affiliations:** 1 Public Health Emergency Management Officer, Bati District Health Office, Bati, Ethiopia; 2 Lecturer, Environmental health department, Debremarkos University, Debre Markos, Ethiopia; 3 Lecturer, Public health Department, Debremarkos University, Debre Markos, Ethiopia; 4 Lecturer, Midwifery Department, Kotebe Metropolitan University, Addis Ababa, Ethiopia; 5 Lecturer, Midwifery Department, Debremarkos University, Debre Markos, Ethiopia

**Correction to: BMC Pediatr. 22, 5 (2022)**

**https://doi.org/10.1186/s12887-021-03072-6**

Following the publication of the original article [1], it has been noticed that author group section has error. Corrected author group section is provided above.

Updated Author contribution section is provided below.

“SA and TM contributed from the inception of study, follow of data collection and analysis of data and preparation of the manuscript. YB had role in data collection, analysis and write-up of the manuscript. All authors read approved the final manuscript. ZD and YT had role in proposal development, analysis and write up and overall supervision of this work.”

The original article has been corrected.
